# Nasal, Oral and Ear Swabs for Canine Visceral Leishmaniasis Diagnosis: New Practical Approaches for Detection of *Leishmania infantum* DNA

**DOI:** 10.1371/journal.pntd.0002150

**Published:** 2013-04-04

**Authors:** Sidney de Almeida Ferreira, Gregório Guilherme Almeida, Soraia de Oliveira Silva, Gabriela Peixoto Vogas, Ricardo Toshio Fujiwara, Antero Silva Ribeiro de Andrade, Maria Norma Melo

**Affiliations:** 1 Departamento de Parasitologia, Instituto de Ciências Biológicas, Universidade Federal de Minas Gerais (UFMG), Belo Horizonte, Brasil; 2 Centro de Desenvolvimento da Tecnologia Nuclear, Comissão Nacional de Energia Nuclear, Campus da Universidade Federal de Minas Gerais, Belo Horizonte, Brasil; Louisiana State University, United States of America

## Abstract

**Background:**

The aim of this study was to evaluate the potential use of nasal, oral, and ear swabs for molecular diagnosis of canine visceral leishmaniasis (CVL) in an endemic urban area in Brazil.

**Methodology/Principal Findings:**

Sixty-two naturally infected and ten healthy dogs were enrolled in this study. Bone marrow aspirates, peripheral blood, skin biopsy, and conjunctival, nasal, oral, and ear swabs were collected. All samples, except blood, were submitted to conventional PCR (cPCR) and quantitative real time PCR (qPCR) to detect and quantify *Leishmania infantum* DNA, respectively. All dogs were submitted to thorough clinical analysis and were included based on a combination of serological (ELISA immunoassay and immunofluorescent antibody test) and parasitological methods. The cPCR positivity obtained from nasal swab samples was 87% (54/62), equivalent to those from other samples (P>0.05). Positive results were obtained for 79% (22/28) in oral swabs and 43% (12/28) in ear swab samples. A significant difference was observed between these data (P = 0.013), and the frequency of positive results from oral swab was equivalent to those from other samples (P>0.05). The use of ear swab samples for cPCR assays is promising because its result was equivalent to skin biopsy data (P>0.05). The qPCR data revealed that parasite loads in mucosal tissues were similar (P>0.05), but significantly lower than the parasite burden observed in bone marrow and skin samples (P<0.05).

**Conclusions:**

Nasal and oral swab samples showed a high potential for the qualitative molecular diagnosis of CVL because their results were equivalent to those observed in samples collected invasively. Considering that mucosae swab collections are painless, noninvasive, fast and practical, the combination of these samples would be useful in massive screening of dogs. This work highlights the potential of practical approaches for molecular diagnosis of CVL and human leishmaniasis infections.

## Author Summary

Visceral leishmaniasis (VL) is an important public health problem in different regions of the world. It presents high lethality in human cases without suitable treatment and is considered one of the most important disorders in dogs, the main domestic reservoir of the etiological agent of VL (*Leishmania infantum*). Most cases of VL in Latin America occur in Brazil, and control campaigns have not shown satisfactory results. The diagnosis of human and canine infection is critical for making decisions regarding surveillance and control policies. In this work, we propose a non-invasive collection method of mucosal and epithelial cells for the molecular diagnosis of canine VL by conventional polymerase chain reaction (cPCR) and for the estimation of parasite load by quantitative real time PCR (qPCR). We used nasal, oral, and ear swabs as practical, simple, painless and fast alternatives for collecting samples. These procedures are according to the need of more simplified methods for detecting *L. infantum* infection by using robust diagnostic techniques such as cPCR and qPCR. Additionally, potential applications for diagnosing human VL are highlighted.

## Introduction

Visceral leishmaniasis (VL) is considered the most severe manifestation among the different clinical expressions of leishmaniasis in humans [1], [2]. This potentially fatal disease is a zoonosis in the Americas [3], and it is caused by *Leishmania infantum* ( = *L. chagasi*).

From a veterinary perspective, the canine VL (CVL) is considered one of the most important diseases in dogs, which represent the main domestic reservoir of parasite and can present risk for human infection [4], [5], [6].

According to the World Health Organization, 3 primary measures should be applied for controlling VL: (i) insecticide-based control of sand flies, (ii) diagnosing and treating human cases, (iii) diagnosing and euthanizing seropositive dogs, although this option is very polemic and disputable [2]. Diagnosis is described in 2 of the 3 recommendations adopted for controlling the disease, demonstrating the strategic importance of proper diagnosis. A correct diagnosis in both humans or dogs is critical because it helps to make decisions more suitable to regions where control measures are more necessary [7]. Furthermore, accurate diagnosis is very desirable for identifying infected animals and avoiding elimination of non-infected dogs.

Various techniques are available for diagnosing CVL infection, which are typically divided into parasitological, immunological, and molecular methods. The parasitological procedures allow the identification of the etiological agent and rely on tissue cultures and cytological or histological analysis based on optical microscopy [8].

The main immunological tests used for diagnosing CVL infection are based on serological methods [9]. In Brazil, enzyme linked immunosorbent assay (ELISA) and immunofluorescence antibody test (IFAT) have been used for CVL surveillance [10]. However, these tests have limitations such as low sensitivity in asymptomatic dogs [11] and cross-reactions with trypanosomiasis and cutaneous leishmaniasis (CL) [12], [13].

The molecular methods are primarily based on polymerase chain reaction (PCR) and have been extensively described for qualitative CVL diagnosis exhibiting high sensitivity, specificity and reproducibility [14], [15], [16]. Specific DNA sequences are easily detected by conventional PCR (cPCR). Quantitative real time PCR (qPCR) is a more recently developed technological approach that permits not only diagnosis but accurate parasite load estimation, and it has been applied for monitoring treatment efficacy [17], [18], [19].

The association of PCR with non-invasive sampling techniques represents a high potential for contributing to CVL diagnosis. Previous studies have described the use and feasibility of the conjunctival swab for detecting *Leishmania* DNA in dogs in Brazil [16] and Italy [20]. Therefore, swabs can be used to easily collect cells from the mucosa and possibly from other anatomical regions of dogs. Thus, the aim of this work was to evaluate oral, nasal, and ear swabs as alternative resources for carrying out qualitative molecular CVL diagnosis by cPCR and estimating parasite burden in these tissues by qPCR. This study is based on the need of more simplified methods for assessing the CVL infections in naturally infected dogs in endemic areas such as Latin America, by using techniques with higher sensitivity and specificity than serological methods used for CVL diagnosis.

## Methods

### Ethics statement

Experiments with dogs were performed in compliance with the guidelines of the Institutional Animal Care and Committee on Ethics of Animal Experimentation (“Comitê de Ética em Experimentação Animal”, national guidelines, Law number 11.794, 8/10/2008) from Universidade Federal de Minas Gerais; approved protocol number: 183/08.

### Dogs

This study was designed in Belo Horizonte, the capital of Minas Gerais State, Brazil, an urban and endemic area that is considered one of the regions most affected by VL in Brazil [21]. Sixty-two naturally infected mongrel dogs of both sexes, unknown ages, and destined for euthanasia were collected in the Municipal Zoonotic Diseases Control Department of Belo Horizonte, MG. Infected animals enrolled in this study showed positive results in ELISA and IFAT, which were performed according to protocols recommended by specific Brazilian legislations. Additionally, inclusion of these dogs was based on a positive result in the parasitological culture test and/or simultaneous positivity of ELISA and IFAT techniques carried out in-house (topic 5). Samples from 10 healthy mongrel dogs of both sexes and free from *Leishmania* infection were used as negative controls and were provided by Federal University of Ouro Preto, MG. All animals were submitted to thorough clinical analysis.

### Clinical samples

Samples were collected in two distinct moments. The first and second collections involved 34 and 28 naturally infected dogs respectively. Seven clinical samples were collected, including nasal, oral, ear, and conjunctival swab, skin biopsy, bone marrow and peripheral blood. All these samples were obtained from all animals (n = 62), except oral and ear swabs. These 2 samples were used only in the second collection (n = 28 dogs). Previously, dogs were anesthetized using 2% xilazine (2.2 mg/kg, Syntec, Brazil) and 2.5% thiopental (9.0 mg/kg, Cristália, Brazil).

Sterile swabs for microbiological isolation (Inlab) were used to remove exfoliative cells from the ear epithelium and nasal, oral, and conjunctival mucosae. A swab was firmly rubbed against the oral mucosa, the inner nasal mucosa in both nostrils, and the lower eyelid of both eyes separately (Figure S1). To collect epithelial cells, a sterile swab was immersed in sterile phosphate-buffered saline and rubbed against the internal surface of the left ear, which had been cleaned with 70% ethanol. Swab tips were broken and transferred into the DNAse-free tubes. Skin biopsies were obtained from the internal surface of the right ear using 5.0-mm sterile punches. Bone marrow aspirates (∼1.0 mL) were collected from the sternum using sterile 10 mL syringes and needles (18 gauges) and divided in 3 fractions. Approximately 200 µL were transferred to DNAse-free tubes for DNA extraction. A drop was added and smeared on a clean slide, and the remaining volume was used for cultures (topic 4). Five milliliters of peripheral blood were collected from the jugular vein. One fraction was transferred into tubes containing ethylenediamine tetraacetic acid (EDTA) for DNA extraction. A second aliquot was stored in a tube without EDTA for obtaining serum. For DNA purification, all samples were immediately kept on ice for transportation and stored at −20°C until use.

### Parasitological tests

The presence of parasites was investigated using optical microscopy with 1000× magnification. Slides smears were stained using the modified Giemsa method (Bioclin, Brazil). Bone marrow aspirates were added to Novy-McNeal-Nicolle medium containing 12% rabbit defibrinated blood and Minimum Essential Medium (GIBCO BRL, USA) containing 10% fetal calf serum (CULTILAB, Brazil), penicillin (100 U/mL), and streptomycin (1.0 µL/mL; GIBCO BRL, Life Technologies USA). Cultures were examined by optical microscopy and subcultured thrice over a 10-day period, after which all culture tubes were reexamined.

### Serological and biochemical tests

Sera from dogs were divided into aliquots and submitted to serological and biochemical tests. ELISA assays were carried out to measure total serum IgG as described elsewhere [22]. IFAT assays were performed based on a standardized protocol [23]. The cut-off value was ≥1∶40, as recommended by the Brazilian legislation. In both serological tests antigens were prepared from cultured *L. infantum* promastigotes, MHOM/BR/1967/BH46 strain.

Some biochemical parameters were measured to complement the clinical analysis. Serum albumin and globulins levels were assessed using Biuret reagent (BIOCLIN, Brazil) at an absorbance of 510 nm (Epoch, Biotek, USA). The colorimetric kinetics method was used to measure serum creatinine (Cobas Mira Classic, Roche, Germany). Finally, serum urea level was assessed using a colorimetric enzymatic assay (BIOCLIN, Brazil).

### DNA extraction

Each swab used in collections was immersed in a lysis buffer solution [50 mM Tris, 50 mM NaCl, and 10 mMEDTA (pH 8.0)], 1% Triton X-100, and proteinase K (250 mg/mL). This mixture was incubated at 56°C for 2 h, eluted from the cotton swab and transferred to 1.5 mL DNAse-free tubes. Then, the phenol-chloroform method was performed as described elsewhere [16]. Purified DNA was suspended in 30 µL of sterile H_2_O.

DNA purification from bone marrow and skin biopsy samples was performed using the Nucleo Spin kit (Macherey-Nagel, Germany) according to the manufacturer's instructions.

### Conventional PCR (cPCR)

To detect *L. infantum* DNA, the following *L. donovani* complex-specific primers were used: [5′ ACG AGG TCA GCT CCA CTC C 3′], [5′ CTG CAA CGC CTG TGT CTA CG 3′]. The cPCR reaction was conducted to amplify the kinetoplast DNA (kDNA) minicircle conserved region of 100 base pairs. For each sample, a master mix of 10 µL was prepared as follows: 1.0 µL of DNA preparation, 5.0 µL of Master Mix Go Taq (Promega, USA) each primer at 1.0 pmol/µL, and ultrapure H_2_O. In all cPCR runs, DNA purified from *L. infantum* at 1.0 ng/µL (MHOM/BR/1967/BH46 strain) and DNA from a recognized infected dog were used as positive controls. DNA extracted from a non-infected dog and water were used as negative controls to assess nonspecific annealing of primers and contamination, respectively. The cPCR reaction was carried out as previously described [24]. The final results were analyzed on a 5% polyacrylamide gel stained using AgNO_3_.

### Real time PCR (qPCR)

Parasite loads were estimated on the basis of absolute quantification using qPCR, as described previously [25], [26]. Primers addressed to the DNA polymerase gene (GenBank accession code AF009147) and canine β-actin gene (GenBank accession code NM_001195845.1) were used [22]. This canine housekeeping gene was adopted as an endogenous control to verify DNA integrity and to normalize the calculations. Standard curves were generated using known amounts of TOPO PCR 2.1 plasmids (Invitrogen, USA) containing cloned canine genes of β-Actin (307 bp) or *L.infantum* DNA polymerase (90 bp). Because these genetic sequences are single-copy genes, the final results were expressed as the number of parasites per canine cells. Reactions were carried out as described previously [22] using the ABI Prism 7500 Sequence Detection System (SDS Applied Biosystems, Foster City, CA, USA).

### Statistical analysis

The frequencies of positive results were compared between paired clinical samples by using the chi-square test or by Fisher's exact test for a number of dogs above or below 30 individuals, respectively. Data distributions were evaluated using the Kolmogorov-Smirnov and D'Agostino-Pearson normality tests. Parasite burdens were compared in pairs using the Mann-Whitney *U* test. The significance level was set at 5%, and the differences were considered significant when the P value <0.05.

## Results

Firstly, the clinical and biochemical analysis indicated that only 6 naturally infected dogs did not present clinical signs and biochemical alterations associated to CVL. These animals were not submitted to statistical analysis separately due to the small sample size.

All healthy control animals were negative for all diagnostic tests confirming the specificity of the techniques used in this study.

### Conventional PCR analysis

According to the qualitative molecular diagnosis data, the frequencies of positive results were as follows: nasal swab, 87% (54/62); conjunctival swab, 76% (47/62); skin biopsy, 81% (50/62); bone marrow biopsy, 90% (56/62). These cPCR results were compared considering paired samples. Based on the qualitative molecular diagnosis data, it was shown that the positivity obtained using nasal swabs was equivalent to those for samples obtained invasively and for conjunctival swab (P>0.05), (Table 1). This last clinical sample showed a frequency of positive results lower than that calculated for bone marrow samples (P = 0.031). Furthermore, the conjunctival swab data was equivalent to skin biopsy data (P>0.05), (Table 1).

**Table 1:** Paired comparisons between cPCR results obtained from different clinical samples in 62 naturally infected dogs.

	*Positivity/sample*	
*Positivity/sample*	Nasal swab*	Conjunctival swab**
	54/62 (87%)	47/62 (76%)
Skin biopsy	P>0.05	P>0.05
50/62 (81%)		
Bone marrow biopsy	P>0.05	P = 0.031
56/62 (90%)		
Conjunctival swab**	P>0.05	-
47/62 (76%)		

* Only the left nostril was considered;

** Only the left conjunctiva was considered.

The *P* values were calculated using the chi square test.

Oral and ear swab samples were collected from only the last 28 naturally infected dogs. Then, the cPCR frequencies of positive results were recalculated for other samples using 28 animals, and statistical analysis was performed. Positive results were as follows: oral swab, 79% (22/28); ear swab, 43% (12/28); nasal swab 75% (21/28); conjunctival swab 54% (15/28); skin biopsy, 68% (19/28); bone marrow biopsy, 79% (22/28) (Tables 2 and 3). The result obtained with oral swab samples was also statistically equivalent to those obtained with skin biopsy and bone marrow samples (P>0.05). Ear swab samples showed low positivity compared to bone marrow samples (P = 0.013). However, the ear swab performance was statistically equivalent to that obtained for skin biopsy (P>0.05), (Table 2).

**Table 2:** Paired comparisons between cPCR results obtained from oral or ear swab and clinical samples obtained invasively.

	*Positivity/sample*	
*Positivity/sample*	Oral Swab	Ear Swab
	(22/28) 79%	(12/28) 43%
Skin biopsy	P>0.05	P>0.05
(19/28) 68%		
Bone marrow biopsy	P>0.05	P = 0.013
(22/28) 79%		

The *P* values were calculated using Fisher's exact test.

**Table 3:** Paired comparisons between cPCR results obtained from different swab samples in 28 naturally infected dogs.

	*Positivity/sample*	
*Positivity/sample*	Oral Swab	Ear Swab
	(22/28) 79%	(12/28) 43%
Nasal swab*	P>0.05	P = 0.029
(21/28) 75%		
Conjunctival swab**	P>0.05	P>0.05
(15/28) 54%		
Ear swab	P = 0.013	-
(12/28) 43%		

* Only the left nostril was considered;

** Only the left conjunctiva was considered;

The *p* values were calculated using Fisher's exact test.

According to the comparisons between clinical samples obtained noninvasively, the frequency of positive results obtained with ear swab samples was lower than that calculated with nasal and oral swab samples (P = 0.029 and P = 0.013, respectively). For all other comparisons, no significant differences were observed between the frequency of positive results (P>0.05), (Table 3).

**Figure 1:**
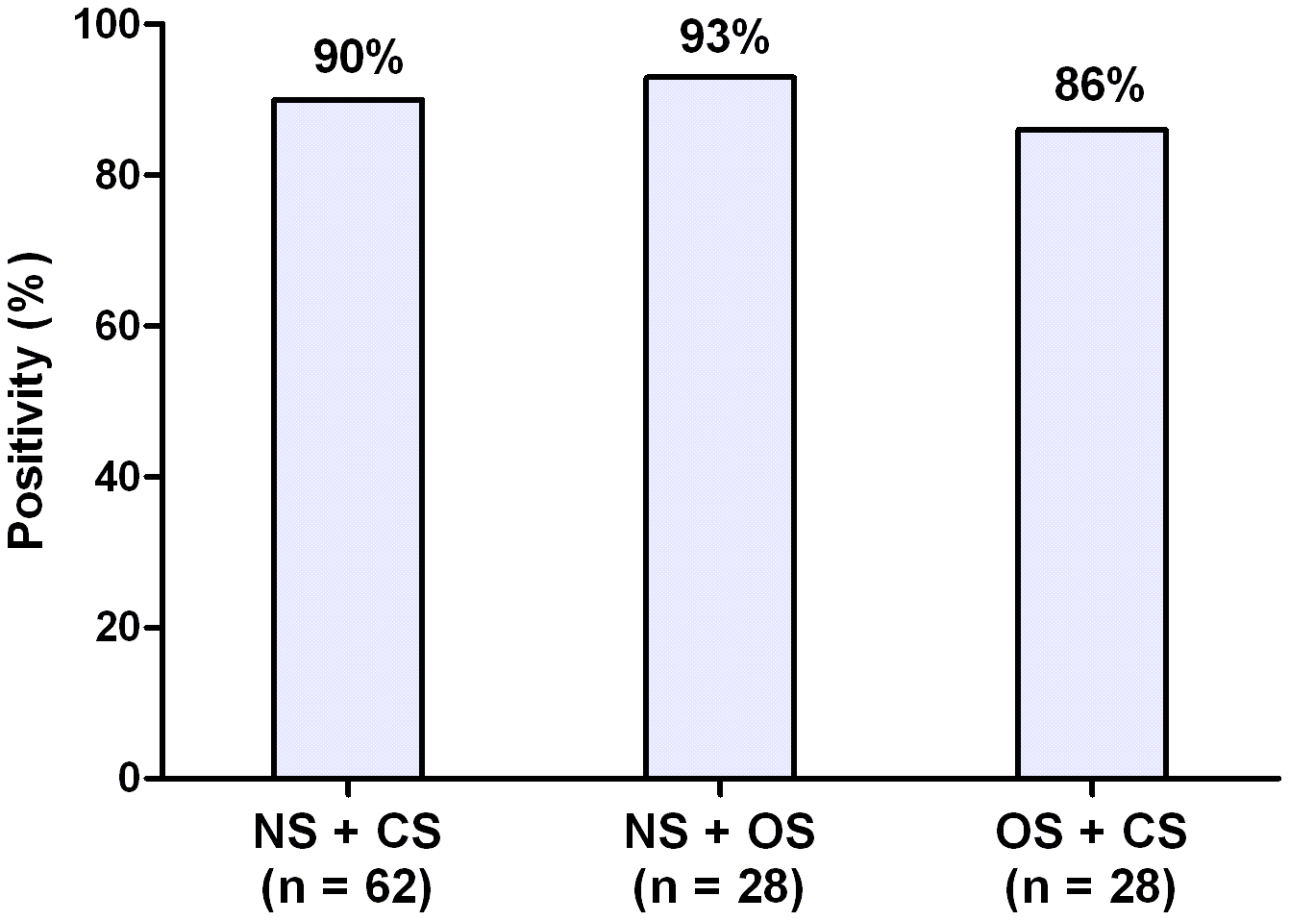
Conventional PCR positivity obtained from combination of clinical samples collected using mucosal swabs. NS: nasal swab; CS: conjunctival swab; OS: oral swab; n: number of naturally infected dogs.

The combination of 2 different mucosal swab samples showed frequencies of positive results as follows: nasal and conjunctival swabs, 90% (56/62); nasal and oral swabs, 93% (26/28); oral and conjunctival swabs, 86% (24/28) (Figure 1).

**Figure 2:**
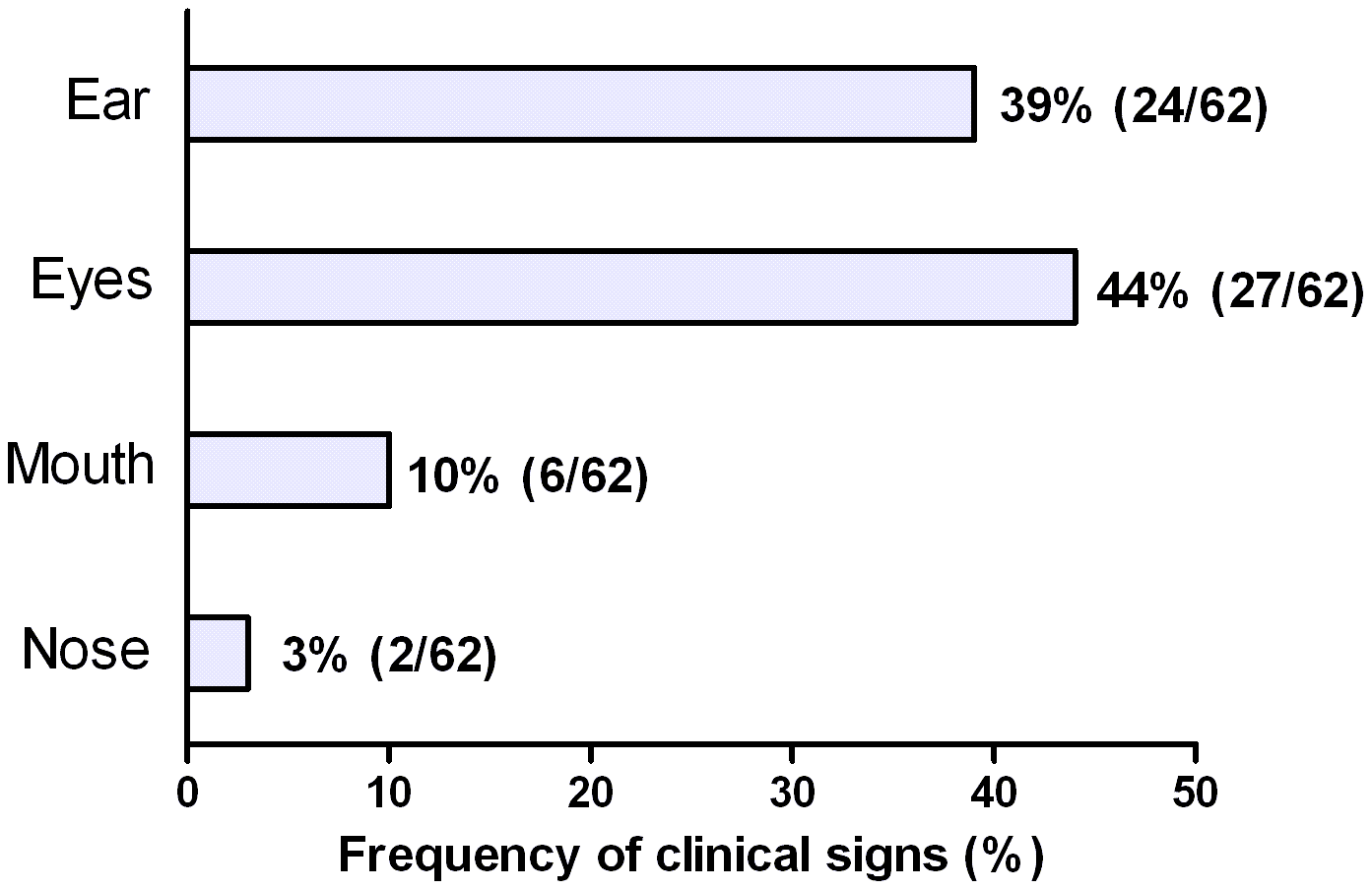
Frequency of clinical signs detected in ear, eyes, mouth and nose in naturally infected dogs.

The frequency of clinical signs observed in sites where swabs were applied was calculated. In all, 39% of dogs (24/62) showed signs on the ear, including exfoliative, nodular, and ulcerative lesions, desquamation, and hyperqueratosis. Signs in the eyes were observed in 44% of dogs (27/62) including uveitis, conjunctivitis, mucosa hyperpigmentation, hyperemia, and keratitis. Ten percent of dogs (6/62) showed signs in the mouth, including ulcers, mucosa hyperpigmentation, and nodules. Finally, only 3% (2/62) of dogs showed clinical signs on the nose, including epistaxis and pustules (Figure 2).

### Real time PCR analysis

**Figure 3:**
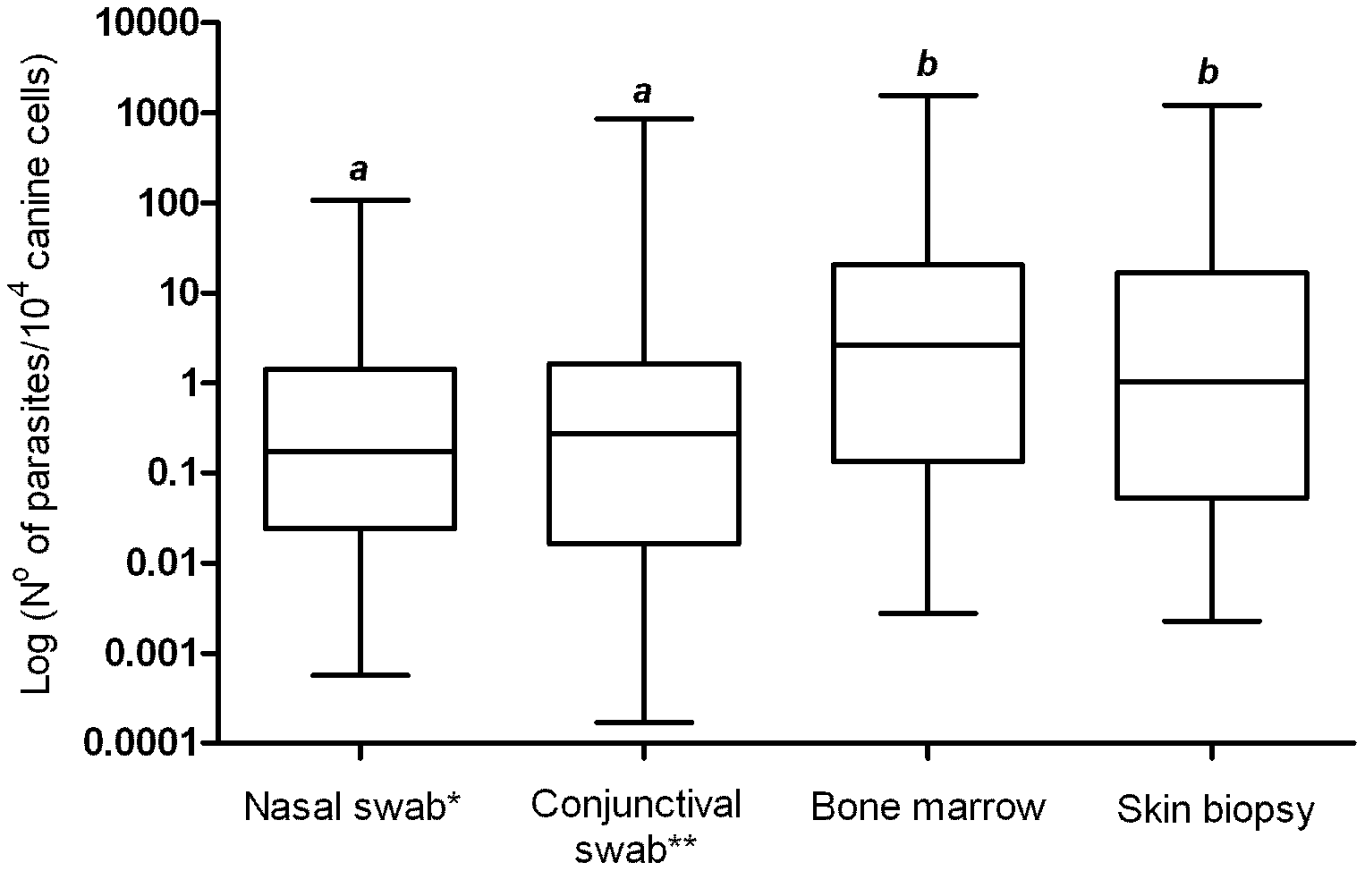
Estimated parasite loads in different clinical samples from 62 naturally infected dogs. Data distributions with distinct letters are significantly different according to the Mann-Whitney *U* test. Nasal swab and bone marrow (P = 0.0004); nasal swab and skin biopsy (P = 0.0074); conjunctival swab and bone marrow (P = 0.0076); conjunctival swab and skin biopsy (P = 0.04). *Only the left nostril was considered. **Only the left conjunctiva was considered.

From a quantitative point of view, parasite burdens were estimated in the nasal and conjunctival swabs, skin biopsy and bone marrow samples from 62 naturally infected dogs. The parasite loads obtained from conjunctival and nasal swab samples were equivalent (P>0.05). On the other hand, the parasitism in the ocular and nasal mucosae was lower than those estimated in the clinical samples obtained invasively (P<0.05). In fact, the highest parasite loads were detected in the bone marrow and skin biopsy, but no difference was observed between these samples (P>0.05) (Figure 3).

**Figure 4:**
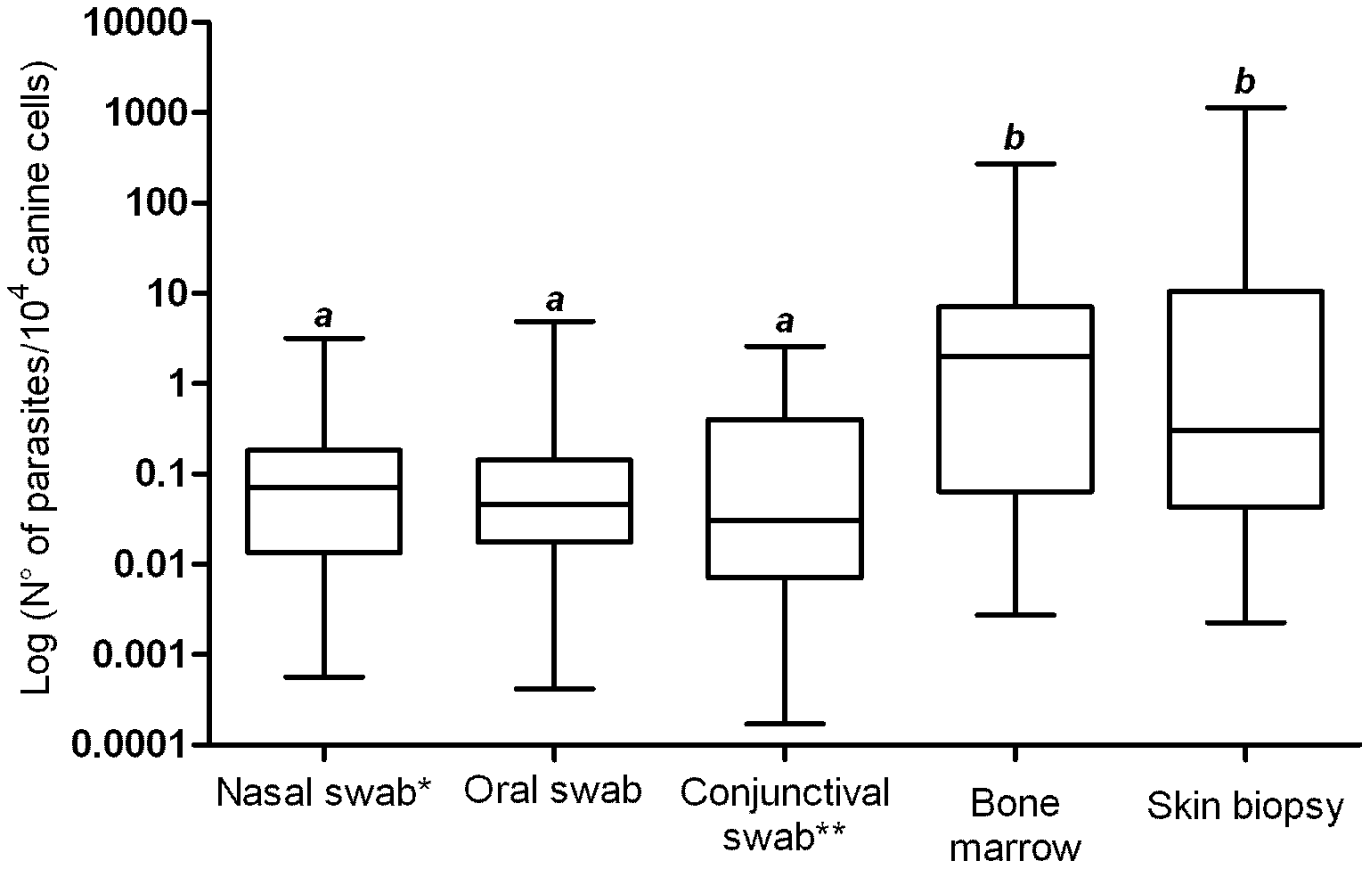
Estimated parasite loads in different clinical samples from 28 naturally infected dogs. Data distributions with distinct letters are significantly different according to the Mann-Whitney *U* test. Nasal swab and bone marrow (P = 0.0024); Nasal swab and skin biopsy (P = 0.019); Oral swab and bone marrow (P = 0.0013); Oral swab and skin biopsy (P = 0.0058); Conjunctival swab and bone marrow (P = 0.0009); Conjunctival swab and skin biopsy (P = 0.0057) *Only the left nostril was considered. **Only the left conjunctiva was considered.

The oral and ear swabs were collected from the last 28 dogs. Then, the parasite loads in other clinical samples, except blood, were recalculated using this sample size. The low parasite burden in mucosae was confirmed, and there was no significant difference among nasal, oral, and conjunctival swab samples (P>0.05). Once more, parasite loads estimated in bone marrow and skin biopsy samples were equivalent (P>0.05). At the same time, the parasitism in these two samples was higher than those estimated for oral, nasal and conjunctival mucosae (P<0.05), (Figure 4). It was not possible to assess the parasite burden in ear swab samples.

## Discussion

According to our study, nasal and oral swabs samples showed a high potential for CVL molecular diagnosis by using cPCR because of their high positive indices, which were equivalent to those obtained from samples collected invasively. CVL is a systemic disease and the infection can occur in a wide variety of organs and tissues [27], [28], [29]. Considering that mucosae undergo high cellular proliferation and a constant generation of exfoliative cells, we focused on these tissues on the basis of swab practicability for collecting biological material.

We used conjunctival swab samples in this work owing to its promising results for CVL molecular diagnosis described previously by our group [16], [22], [30], [31]. Thus, the conjunctival swab sample was adopted as a reference sample collected noninvasively to compare with nasal, oral and ear swab samples.

Conjunctival and nasal swab samples were collected separately from both eyes and nostrils, respectively, and treated as distinct samples. The simultaneous use of 2 ocular swabs and 2 nasal samples increased the positivity of cPCR (data not shown) and is highly recommended for screening dogs. Nonetheless, there was no significant difference between the cPCR positive results calculated for right and left nostrils or conjunctivas (data not shown).

Although the conjunctival swab positivity has been considered lower than that obtained from bone marrow, the combination of ocular samples provided a diagnostic result statistically equivalent to the samples obtained invasively (data not shown). Anyway, in all paired comparisons, just one conjunctiva and one nostril was adopted in order to avoid undue favoritism for these swab samples. This study demonstrated *Leishmania* DNA detection from nasal swabs for the first time. This method increases the number of available options for detecting parasites in dogs by using cPCR.

Considering that amastigotes within macrophages can reach the mucosae through the lymphatic and/or hematogenous route [32] nasal tissues are susceptible to parasite colonization owing to the large number of blood vessels in the mucosa. Furthermore, the muzzle is a favorable site for sand fly bites due to the absence of hair. The DNA yield obtained from nasal swabs was markedly high (data not shown), demonstrating that these samples are rich source of DNA for molecular biology assays.

As described for the nasal swab samples, oral swabs yielded an equivalent result compared to the samples collected invasively for diagnosis using cPCR. Particularly, the frequency of positive results was high in the bone marrow because parasites naturally migrate to lymphoid tissues [33]. Interestingly, the frequency of positive results obtained from oral swab samples was the same as that calculated for bone marrow samples. Thus, oral mucosa may be a more practical choice for collecting samples to be used for qualitative diagnosis by using PCR techniques.

In our study, oral swab samples were used for molecular diagnosis of CVL for the first time in a Brazilian endemic region, and we confirmed a high potential for detecting DNA from *L. infantum*. This clinical sample was firstly evaluated in an endemic region from Italy using qPCR assays, but it showed low sensitivity for detecting *Leishmania* DNA in seropositive dogs [20]. According to these authors, the presence of *Leishmania* DNA in oral swabs may have implications on the transmission of parasites among dogs through licking and bites. Parasite transmission to dogs in the absence of phlebotomines has been confirmed through blood donation [34], placenta (vertical transmission) [35], [36], and sex (venereal transmission) [37]. However, transmission through licking, bites, and wounds is still unproven [9]. Further studies to confirm this hypothesis are necessary.

Various pathological processes associated with CVL have been detected in conjunctival, nasal, and oral mucosae. Nodular lesions, granulomatous, lymphoplasmacytic, and pyogranulomatous manifestations have been well described in these tissues [38], [39], [40]. In the context of CVL diagnosis, the use of swabs has been indicated for collecting samples particularly in the presence of dermatological lesions [41]. However, according to our data, most dogs had no lesions in conjunctival, nasal, or oral mucosae, and the *L. infantum* DNA was detected in these tissues with a high positivity. This result emphasizes the potential of these clinical samples, which were obtained using swabs for diagnosing the infection in dogs without clinical signs.

In cPCR experiments, the ear swab presented low positivity. DNA extracted from this sample showed poor yield and purity. The absorbance measured using spectrophotometer indicated protein contamination in many samples (data not shown), and the low quality of DNA likely affected cPCR and qPCR performances. On the other hand, the cPCR positive index obtained from ear swab samples was equivalent to that calculated for skin biopsy samples. This result is promising, and the DNA extraction from ear swab should be improved. In addition, ear swab should be evaluated in further studies, and it can be a more attractive choice to avoid the use of invasive methods such as skin scraps and biopsies for the molecular diagnosis of CVL.

High frequencies of positive results were obtained using a combination of samples collected using swabs. This is particularly useful for screening dogs in large-scale studies. Sensitive methods are indicated to preliminary surveys for diagnosing infections in populations, and the sensitivity of the diagnostic techniques is one of the accuracy measures of interest to public health policymakers [42]. According to our results and considering the practicability of mucosae swabs, we strongly recommend the combination of these clinical samples for diagnosing CVL based on PCR assays. In this case, the swab samples could be mixed and processed as a unique sample in order to enhance the diagnostic sensitivity. Additionally, this procedure may simplify sample management and save time, permitting the analysis of a large number of dogs.

We also analyzed parasitism levels in different tissues. Generally, parasite loads in mucosae were low indicating weak parasite colonization. Besides, bone marrow and skin biopsy samples showed high and similar parasite burdens. These results were the same when we separately compared parasitism in these different clinical samples in 62 and 28 dogs, thus reinforcing our conclusions.

Parasites show natural tropism towards lymphoid tissues, and different studies have shown that bone marrow is a good source for *Leishmania* DNA detection and quantification in agreement with our study [43], [44], [45]. The high parasite load in the skin is an important characteristic that helps to explain the role of dogs as parasite reservoirs in endemic regions [22], [46], [47]. Therefore, we chose to examine the canine skin. As a matter of fact, it has been suggested that ear tissue should be used as the primary site for parasitological confirmation in dogs [48]. In addition, it was pointed as the better anatomical region from skin to perform biopsies and PCR assays for detection of *Leishmania* infection [49].

In our experimental context, there was no difference among nasal, oral, and conjunctival swab samples in detecting *Leishmania* infection in naturally infected dogs. All of these tissues permitted the evaluation of parasite load using qPCR. These results are relevant because distribution of parasites is not uniform in host organs and tissues [50]. Thus, combining different canine clinical samples would be useful for obtaining more conclusive diagnostic results by using PCR. Choosing appropriate samples for detecting parasites in different affected tissues of dogs is necessary for accurate diagnosis and/or prognosis. This is very important for the orientation of clinicians' work [41]. Hence, we recommend the use of nasal, oral, and conjunctival swabs on the basis of their practicability and potential for PCR assays.

The use of swabs has several applications in the molecular diagnosis of human leishmaniasis as well. In Ecuador, 15 of 16 patients presenting CL tested positive by using lesion swabs and PCR [51]. In Colombia, human CL was diagnosed by combining PCR with conjunctival and nasal swabs [52]. Additionally, oral swab was used to successfully detect VL infection in patients from India [53]. These data extend the potential of these clinical samples for VL and CL molecular diagnosis.

In summary, we demonstrated the utility of nasal, oral, and ear swab samples for detecting *Leishmania* infection in dogs. Because these collections are painless, noninvasive, fast, and practical, combining these samples would be useful for screening large numbers of dogs. Large-scale evaluations in the field considering epidemiological aspects and infected dogs without clinical signs should be conducted.

## Supporting Information

Figure S1 **Nasal (A), oral (B), ear (C), and conjunctival swab (D) collection.**
(TIF)

Figure S2 **Flowchart of the experimental design.**
(DOC)

Table S1 **STARD checklist for reporting studies of diagnostic accuracy.**
(DOC)
