# Supplementary figures and images for: Nasal, Oral and Ear Swabs for Canine Visceral Leishmaniasis Diagnosis: New Practical Approaches for Detection of Leishmania infantum DNA
Source: PLoS Negl Trop Dis. 2013 Apr 4;7(4):e2150. doi: 10.1371/journal.pntd.0002150 (PMC3617150; doi:10.1371/journal.pntd.0002150)

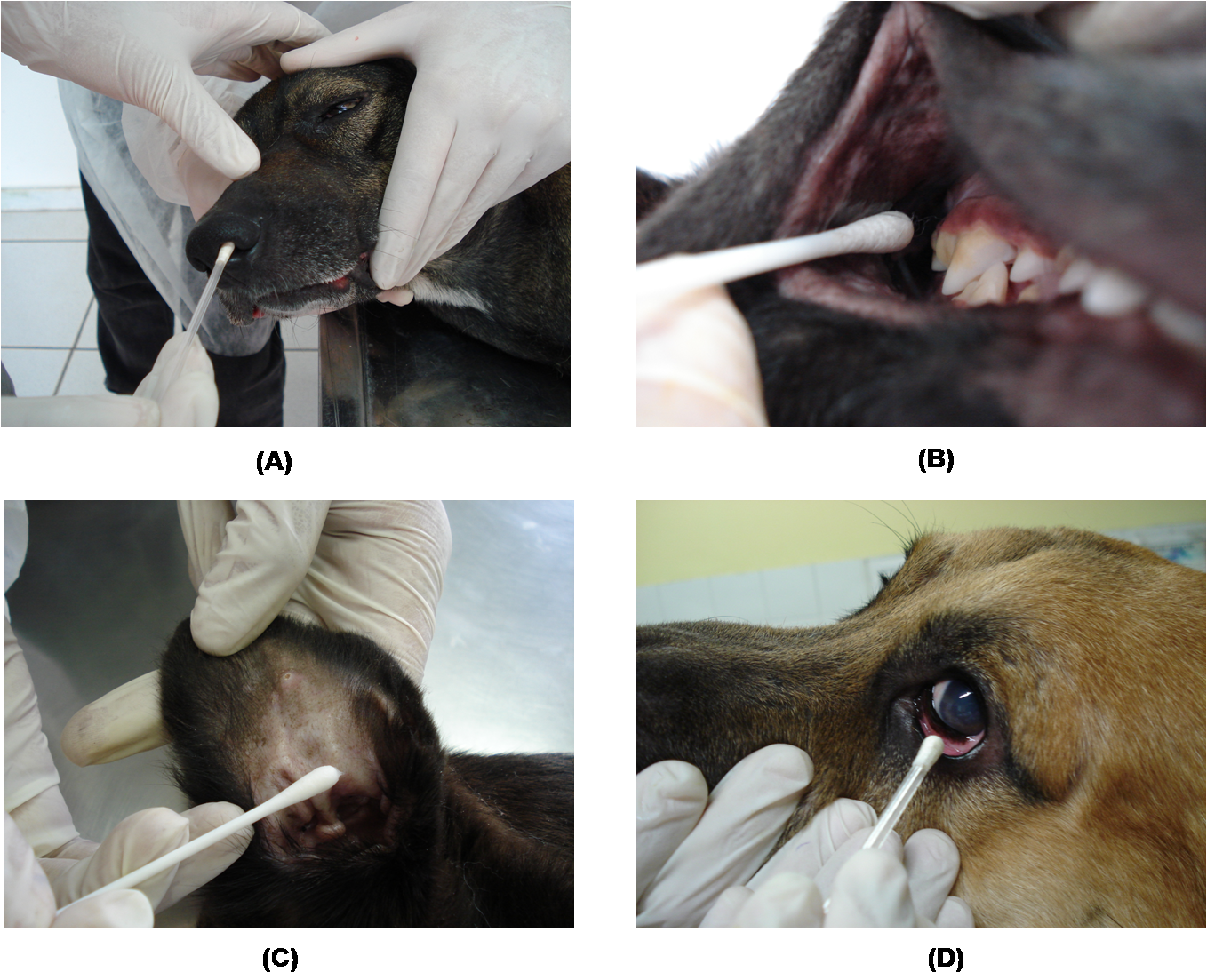

Supplement: Figure S1 — Nasal (A), oral (B), ear (C), and conjunctival swab (D) collection. (TIF) [file pntd.0002150.s001.tif]
