# Supplementary material for: Nasal, Oral and Ear Swabs for Canine Visceral Leishmaniasis Diagnosis: New Practical Approaches for Detection of Leishmania infantum DNA
Source: PLoS Negl Trop Dis. 2013 Apr 4;7(4):e2150. doi: 10.1371/journal.pntd.0002150 (PMC3617150; doi:10.1371/journal.pntd.0002150)
Supplement: Figure S2 — Flowchart of the experimental design. (DOC) [file pntd.0002150.s002.doc]

Eligible dogs

n = 62 (analysis of nasal swab)

n = 28 (analysis of oral and ear swabs)

Excluded dogs

n = none

Abnormal result

n = none

Index test

n = 62 (analysis of nasal swab)

n = 28 (analysis of oral and ear swabs)

Normal result

n = 62 (analysis of nasal swab)

n = 28 (analysis of oral and ear swabs)

Inconclusive result

n = none

Inconclusive

n = none

Target condition present

n = See tables 1, 2 and 3

Target condition absent

n = See tables 1, 2 and 3

**Figure S2:** Flowchart of the experimental design.
